# Excess heat capacity and entropy of mixing along the hydroxyapatite-chlorapatite and hydroxyapatite-fluorapatite binaries

**DOI:** 10.1007/s00269-021-01167-1

**Published:** 2021-10-28

**Authors:** Edgar Dachs, Artur Benisek, Daniel Harlov, Max Wilke

**Affiliations:** 1 Fachbereich Chemie und Physik der Materialien, Universität Salzburg, Jakob-Haringerstrasse 2a, 5020 Salzburg, Austria; 2 Deutsches GeoForschungsZentrum-GFZ, Telegrafenberg, 14473 Potsdam, Germany; 3 Faculty of Earth Resources, China University of Geosciences, Wuhan, 430074 China; 4 Department of Geology, University of Johannesburg, P.O. Box 524, Auckland Park, 2006 South Africa; 5 Department of Earth Sciences, University of Potsdam, 14469 Potsdam, Germany

**Keywords:** Heat capacity, Excess entropy, Hydroxyapatite-fluorapatite binary, Hydroxyapatite-chlorapatite binary, Mixing properties, Standard third-law entropy

## Abstract

The heat capacity, *C*_p_, of synthetic hydroxyapatite [Ca_5_(PO_4_)_3_OH–OH-Ap], as well as of ten compositions along the OH-Ap-chlorapatite (Cl-Ap) join and 12 compositions along the OH-Ap-fluorapatite (F-Ap) join have been measured using relaxation calorimetry (heat capacity option of the Physical Properties Measurement System—PPMS) and differential scanning calorimetry (DSC) in the temperature range of 5–764 K. Apatites along the Cl-OH and F-OH joins were synthesized at 1100 °C and 300 MPa in an internally heated gas pressure vessel via an exchange process between synthetic fluorapatite or chlorapatite crystals (200–500 μm size) and a series of Ca(OH)_2_-H_2_O solutions with specific compositions and amounts relative to the starting apatite. The standard third-law entropy of OH-Ap, derived from the low-temperature heat capacity measurements, is *S*° = 386.3 ± 2.5 J mol^−1^ K^−1^, which is ~ 1% lower than that resulting from low-temperature adiabatic calorimetry data on OH-Ap from the 1950’s. The heat capacity of OH-Ap above 298.15 K shows a hump-shaped anomaly centred around 442 K. Based on published structural and calorimetric work, this feature is interpreted to result from a monoclinic to hexagonal phase transition. Super ambient *C*_p_ up to this transition can be represented by the polynomial: $C_{p}^{{\text{OH - Ap}}} {}_{{298K - 442K}}\left( {{\text{J mol}}^{ - 1} {\text{K}}^{- 1}} \right) = {1013.7-13735.5T^{{ - 0.5}}} + 2.616718\,10^{7} T^{{ - 2}} - 3.551381\,10^{9} T^{{ - 3}} .$. The DSC data above this transition were combined with heat capacities computed using density functional theory and can be given by the *C*_p_ polynomial: $C_{p}^{{\text{OH - Ap}}} {}_{{ >\,442K}}\left( {{\text{J mol}}^{ - 1} {\text{K}}^{- 1}} \right) = {877.2-11393.7 T^{ - 0.5}} + {5.452030\,10^{7}} \,T^{- {2}} - {1.394125\,10^{10}} \,T^{- {3}}$. Positive excess heat capacities of mixing, ∆*C*_p_^ex^, in the order of 1–2 J mol^−1^ K^−1^, occur in both solid solutions at around 70 K. They are significant at these conditions exceeding the 2σ-uncertainty of the data. This positive ∆*C*_p_^ex^ is compensated by a negative ∆*C*_p_^ex^ of the same order at around 250 K in both binaries. At higher temperatures (up to 1200 K), ∆*C*_p_^ex^ is zero within error for all solid solution members. As a consequence, the calorimetric entropies, S_cal_, show no deviation from ideal mixing behaviour within a 2σ-uncertainty for both joins. Excess entropies of mixing, ∆S^ex^, are thus zero for the OH-Ap–F-Ap, as well as for the OH-Ap–Cl-Ap join. The *C*_p_–*T* behaviour of the OH-Ap endmember is discussed in relation to that of the F- and Cl-endmembers.

**Supplementary Information:**

The online version contains supplementary material available at 10.1007/s00269-021-01167-1.

## Introduction

Apatite [Ca_10_(PO_4_)_6_(F,Cl,OH)_2_] is the most common and widespread phosphate mineral in the Earth’s crust and lithospheric mantle. In its most common form as fluorapatite [Ca_10_(PO_4_)_6_F_2_], it also is a major mineralogical sink for F and to a lesser extent Cl. As a member of the apatite supergroup, apatite can incorporate a wide range of elements into its structure, some of the more important being C (as CO_3_), Na, S (as SO_4_), Si, Fe, Pb, REE, Th, and U (Hughes and Rakovan 2015). As a major host for F, Cl, and OH, apatite in equilibrium with another major halogen carrier, biotite, can be used as a halogen exchange thermometer (Zhu and Sverjensky 1992; Sallet 2000). It can also be used as a F, Cl, and OH barometer in equilibrium with a fluid (Zhu and Sverjensky 1991). Hence, there is an underlying importance in understanding the thermodynamics of F, Cl, and OH mixing on the halogen site in apatite. To this end an earlier study measuring the *C*_p_ with regard to F-Cl mixing on the halogen site over a broad temperature range has been done by Dachs et al. (2010a). These authors found that F-Cl mixing does not deviate significantly from ideality within a 2σ uncertainty (calorimetric work extant on fluorapatite and chlorapatite endmembers is discussed in that paper and will not be repeated here). This current study continues and completes this earlier study by measuring *C*_p_ with the aim to constrain F-OH and Cl-OH mixing on the halogen site. While no heat capacity data exist for these joins so far, *C*_p_ of synthetic hydroxyapatite has been measured by Egan et al. (1951a) below room temperature down to 13 K, and by Palkin et al. (1991) in the range 55–300 K. Above ambient *T*, Egan et al. (1950) provided heat-content data for this endmember up to 1500 K and Suda et al. (1995) measured *C*_p_ of OH-Ap between 440 and 500 K using DSC techniques. The latter authors demonstrated calorimetrically that there is a structural monoclinic to hexagonal phase transition at around 480 K in this phase as already proposed by van Rees et al. (1973) from birefringence measurements on heated synthetic single OH-Ap crystals.

## Experimental method

### Sample synthesis and characterization

Synthesis of apatites along the F-OH and Cl-OH joins was carried out using synthetic endmember fluorapatite and chlorapatite crystals in the 200–500 micron size range. The fluorapatite and chlorapatite crystals were synthesized according to the technique laid out in Schettler et al. (2010).

The synthesis technique used here consisted of placing 400 mg of either fluorapatite or chlorapatite crystals plus varying amounts of Ca(OH)_2_ + H_2_O in a 5 mm diameter, 4 cm long Pt tube. Once loaded, the Pt tube was arc-welded shut, left up over night in a 100 °C oven to check the seals, and then taken up to 1000 or 1100 °C and 400 MPa in an internally heated gas pressure vessel using Ar as the pressure medium. The synthesis runs were left up from 3 to 6 days. The temperature was measured with 3 S-type thermocouples and calibrated based on measurements of the melting points of NaCl at 843 °C/200 MPa and 904 °C/500 MPa (Akella et al. 1969). The accuracy of the temperature is about ± 5 °C at 200 MPa and ± 20 °C at 500 MPa. Maximum thermal gradients along the capsules were ± 10 °C. Pressure measurement was done with a strain gauge and is accurate to ± 7 MPa for experiments up to 500 MPa. During the experiment, pressure was controlled automatically within ± 5 MPa using the hydraulic system of the intensifier and a programmable control unit. The samples were heated isobarically with a rate of 30 °C min^− 1^ and quenched isobarically with quench rates of 150–200 °C min^− 1^.

During the experimental run the following exchange reactions (not balanced) took place between the fluorapatite or chlorapatite crystals and the Ca(OH)_2_-H_2_O solution via a coupled dissolution-reprecipitation reaction (see Putnis 2009):

$${\text{Ca}}\left( {{\text{OH}}} \right)_{{2}} + {\text{ H}}_{{2}} {\text{O }} + {\text{ Ca}}_{{{1}0}} \left( {{\text{PO}}_{{4}} } \right)_{{6}} {\text{F}}_{{2}} = {\text{ Ca}}_{{{1}0}} \left( {{\text{PO}}_{{4}} } \right)_{{6}} \left( {{\text{F}},{\text{OH}}} \right)_{{2}} + {\text{ CaF}}_{{2}} + {\text{ Ca}}\left( {{\text{OH}}} \right)_{{2}} + {\text{ H}}_{{2}} {\text{O }} + {\text{ HF}}$$

and

$${\text{Ca}}\left( {{\text{OH}}} \right)_{{2}} + {\text{ H}}_{{2}} {\text{O }} + {\text{ Ca}}_{{{1}0}} \left( {{\text{PO}}_{{4}} } \right)_{{6}} {\text{Cl}}_{{2}} = {\text{ Ca}}_{{{1}0}} \left( {{\text{PO}}_{{4}} } \right)_{{6}} \left( {{\text{Cl}},{\text{OH}}} \right)_{{2}} + {\text{ CaCl}}_{{2}} + {\text{ Ca}}\left( {{\text{OH}}} \right)_{{2}} + {\text{ H}}_{{2}} {\text{O }} + {\text{ HCl}}$$

After quench, the Pt capsule was opened and the contents washed 3 or 4 times in nano-pure distilled H_2_O to remove any excess Ca(OH)_2_ or resulting HCl, HF, CaCl_2_, or CaF_2_. The composition of the resulting F-OH apatite or Cl-OH apatite was determined via single crystal XRD of several randomly selected crystals from each of the synthesis runs (cf. Hughes et al. 2016, 2018). Since OH cannot be directly measured by electron microprobe analysis but rather must be calculated via charge balance based on relatively approximate electron microprobe analytical measurements of F or Cl on the halogen site, single crystal XRD was considered the most accurate way of determining the OH vs. F or OH vs. Cl content for each of the apatite syntheses. Wet chemical analysis was also not considered due to the limited amount of each synthesis produced (< 400 mg) and the fact that other portions of each synthesis were being utilized for other purposes such that there was not enough material left over for an accurate wet chemical analysis.

The hydroxylapatite (APS-88) was synthesized by placing 400 mg of commercial hydroxylapatite (SIGMA calcium phosphate tribasic, Batch # 012K1603) plus 100 mg H_2_O in a 5 mm diameter, 4 cm long Pt tube, which was arc-welded shut, and then taken up to 1100 °C and 400 MPa for 72 h in the internally heated gas pressure apparatus utilizing the same technique as outlined above.

Powder-XRD patterns on OH-Ap were measured with a Bruker D8 Advance X-ray diffractometer and used to derive the lattice parameters of synthetic OH-Ap with the programme UnitCell (Holland and Redfern 1997).

### Calorimetric methods

Low-temperature heat capacities were measured with a commercially designed relaxation calorimeter (heat capacity option of the Physical Properties Measurement System (PPMS), constructed by Quantum Design^®^) at Salzburg University. The data were collected three times at 60 different temperatures between 5 and 300 K on samples weighing between 10 and 15 mg (the mean of the three measurements per temperature step is given in supplementary Table S1). For measurement, the sample powders were wrapped in thin Al-foil and pressed to a flat pellet that was then placed on the sample-platform of the calorimeter. A measure of the quality of the thermal conductance between the sample and the sample-platform during a PPMS measurement is the so called sample-coupling. The closer this value is to 100%, the more reliable is the measured heat capacity value. Bad sample couplings, on the other hand, may indicate underestimated *C*_p_’s (see e.g., Dachs and Bertoldi 2005, or Dachs et al. 2010a, for more details. More details on the calorimetric technique, as well on its precision and accuracy can be found in Lashley et al. (2003), Dachs and Bertoldi (2005), or Dachs and Benisek (2011) and will not be repeated here.

Heat capacities between *T* = 282 and 764 K were collected with a Perkin Elmer Diamond differential scanning calorimeter^®^ (DSC). The measurements were performed under a flow of Ar gas and with the calorimeter block kept at *T* = 243.3 K using a Perkin Elmer Intracooler. The heat flow data were collected in temperature intervals of 50 K. Each interval consisted of a temperature scan using a heating rate of 10 K min^− 1^ and isothermal periods 2 min before and after the temperature scan. Each complete measurement included three runs: a blank run, a reference run, and a sample run. Before each sample run, the DSC was calibrated with a reference run using a synthetic single crystal of corundum whose heat capacities were taken from the National Bureau of Standards Certificate (Ditmars et al. 1982). DSC measurements were repeated 3–4 times. The resulting mean and standard deviation of *C*_p_ for a sample are given in Supplementary Table S1. More details on the DSC method applied can be found in Dachs and Benisek (2011).

### Evaluation of the calorimetric data

We have adopted the fitting equation of Boerio-Goates et al. (2002) to fit the PPMS *C*_p_ data of OH-Ap (corresponding Mathematica code is available upon request from the first author):

1 $$C_{p} = {3}R(m\,D(\Theta_{D} ) + n\,E(\Theta_{E} ) + {\text{s}}\,S(\Theta_{S} )),$$

where *D*(Θ_*D*_), *E*(Θ_*E*_), and *S*(Θ_*S*_) are Debye, Einstein, and Schottky functions, respectively, defined as:

2 $$D(\theta_{D} ) = 3\left( {\frac{T}{{\theta_{D} }}} \right)^{3} \int\limits_{0}^{{{\raise0.7ex\hbox{${\theta_{D} }$} \!\mathord{\left/ {\vphantom {{\theta_{D} } T}}\right.\kern-\nulldelimiterspace} \!\lower0.7ex\hbox{$T$}}}} {\frac{{x^{4} e^{x} }}{{(e^{x} - 1)^{2} }}dx}$$

3 $$E(\theta_{E} ) = \frac{{\left( {\frac{{\theta_{E} }}{T}} \right)^{2} e^{{\frac{{\theta_{E} }}{T}}} }}{{\left( {e^{{\frac{{\theta_{E} }}{T}}} - 1} \right)^{2} }}$$

4 $$S(\theta_{S} ) = \frac{{\left( {\frac{{\theta_{S} }}{T}} \right)^{2} e^{{ - \frac{{\theta_{S} }}{T}}} }}{{\left( {1 + e^{{ - \frac{{\theta_{S} }}{T}}} } \right)^{2} }}$$

and *m*, *n*, *s*, Θ_*D*_, Θ_*E*_, and Θ_*S*_ are adjustable parameters (Θ_*D*_, Θ_*E*_, and Θ_*S*_ are the Debye, Einstein and Schottky temperatures, respectively; *m*, *n*, and *s* are weighting factors that also model the *C*_p_ − *C*_v_ difference). An optimal representation of the *C*_P_-*T* data occurred when the data were split into a low and high-temperature segment. Accordingly, two sets of fitting coefficients were obtained using the software Mathematica^®^ (Table 1). The high-temperature *C*_P_ data of OH-Ap were fitted to a polynomial of the form:

5 $$C_{P} = k_{0} + k_{1} T^{ - 0.5} + k_{2} T^{ - 2} + k_{3} T^{ - 3} ,$$

as proposed by Berman and Brown (1985).

**Table 1:** Parameters of Eq. (1), used to calculate smoothed values of the heat capacities of OH-, Cl- and F-apatite endmembers at temperatures between 0 and 298.15 K

Apatite endmember	*T* range [K]	*θ*_D_ [K]	*M* [mol]	*θ*_E_ [K]	*n* [mol]	*θ*_S_ [K]	*s* [mol]
OH-Ap	< 38.86	521.511	23.541	172.731	1.113	102.137	0.443
	38.86–298.15	482.187	13.814	1046.084	7.517	167.088	2.675
Cl-Ap	< 35.44	293.848	7.219	104.261	0.561	24.677	0.004
	35.44–298.15	430.910	13.230	1012.226	7.067	142.943	2.250
F-Ap	< 37.98	352.180	8.701	132.378	0.872	64.644	0.076
	37.98–298.15	490.207	14.252	1209.184	7.214	163.487	2.764

The parameter values for OH-Ap are from this study, those for Cl- and F-Ap from Dachs et al. (2010a)

Compared to the endmember OH-Ap, the PPMS and DSC data measured on the solid solution apatites were treated differently, using the Mathematica function^®^ *Interpolation* to represent their heat capacities as function of temperature.

The calorimetric molar entropies at 298.15 K, *S*^298^, were calculated by solving the integral in Eq. (6) using the Mathematica^®^ function *NIntegrate* (assuming *S*^*T*=0 K^ = 0) and either Eq. (1), in the case of OH-Ap, or *Interpolation* for calculating *C*_P_:

6 $${S}_{cal}={S}^{T=298.15K}- {S}^{T=0K}= \underset{0}{\overset{298.15}{\int }}\frac{{C}_{p}}{T}dT$$

*S*_cal_ corresponds to the standard-state (third-law) entropy, *S*^o^, in the case of endmember OH-Ap. Errors in *S*_cal_ were estimated according to Dachs and Benisek (2011). Due to the low absolute values of *C*_p_ below 5 K, the entropy increment from 0 to 5 K is < 0.02 J mol^−1^ K^−1^ and could be neglected in the determination of *S*_cal_.

### Computational methods using density functional theory (DFT)

Quantum–mechanical calculations were based on the DFT plane-wave pseudopotential approach implemented in the CASTEP code (Clark et al. 2005) included in the Materials Studio software from Biovia^®^. The calculations used the local density approximation (LDA) for the exchange–correlation functional (Ceperley and Alder 1980). To describe the core-valence interactions, norm-conserving pseudopotentials were used with the 1s^1^, 2s^2^2p^4^, 3s^2^3p^3^, and 3s^2^3p^6^4s^2^ electrons explicitly treated as valence electrons for H, O, P, and Ca, respectively. The k-point sampling used a Monkhorst–Pack grid (Monkhorst and Pack 1976) with a spacing of 0.06 Å^−1^. The structural relaxation was calculated by applying the BFGS algorithm (Pfrommer et al. 1997), where the maximum force on the atom was within 0.01 eV/Å. On such relaxed structures, lattice dynamical calculations were performed using the linear response approximation. The transformation of CASTEP heat capacities at constant volume into heat capacities at constant pressure was done as outlined in Benisek and Dachs (2018).

Constructing a structural model for OH apatite for the quantum–mechanical calculation is not straightforward because of the disorder of the OH group. The F atom in apatite is positioned at the special crystallographic position (0, 0, ¼). However this is not the case with the OH group. In the real crystal, OH is disordered above and below this special position (Hughes et al. 1989). Strictly speaking, the mirror plane at ¼ is destroyed. Phonon calculations of a cell, which reflects such disorder, are highly time consuming and were not performed here. For the sake of simplicity, we put the centre of the oxygen and hydrogen at (0, 0, ¼) having a distance of ca. 1 Å between them and let the geometry relaxation find their end-positions. Such a model does not reflect reality and may cause errors in the calculated heat capacities, especially at low temperatures. However, we used the calculated heat capacities only for extrapolation purposes at high temperatures, where such details should not cause significant errors. CASTEP input and output files for our DFT calculation on OH-Ap are available upon request.

## Results

The sample couplings in the PPMS measurements ranged between 99.0–99.4% around room temperature, increased with falling temperature to a maximum between 99.5% and 99.8% around 150 K, and then decreased to values between 95 and 98% at the lowest temperatures. These sample-coupling values indicate good *C*_p_ determinations via the PPMS.

The agreements between PPMS-measured and DSC-measured heat capacity at room temperature were better than ± 0.6% in most cases (with one exception where the difference was 1.2%).

### Heat capacity and standard entropy of hydroxyapatite

The XRD pattern obtained on OH-Ap (sample APS-88) showed only reflections that could be attributed to this phase, no extra peaks steming from possible impurities were detected. Using a hexagonal cell, the lattice constants are a_o_ = 9.4195(4) Å and c_o_ = 6.8865(6) and the cell volume is 529.16(6) Å^3^. This agrees reasonably well with the values determined e.g. by Suda et al. (1995), i.e., a_o_ = 9.4187(5) Å and c_o_ = 6.8805(2) Å, or lattice parameters cited in Tacker and Stormer (1989) for OH-Ap, i.e., a_o_ = 9.422 Å and c_o_ = 6.883 Å.

**Fig. 1:**
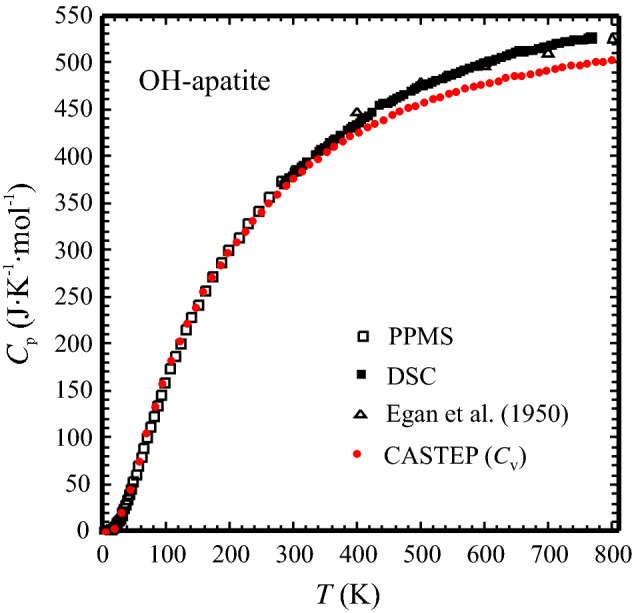
Heat capacity of synthetic hydroxyapatite as function of temperature. Open squares: PPMS data, filled squares: DSC data. The drop-(solution) calorimetry data of Egan et al. (1950) are shown as open triangles. The low-*T*AC data of Egan et al. (1951a) and Palkin et al. (1991) are not plotted to avoid symbol overlap, but their deviation to the PPMS data is shown in Fig. 2. Red dots are heat capacities at constant volume, *C*_v_, of hydroxyapatite, computed with density functional methods (CASTEP). Error bars are smaller than the symbol size

**Fig. 2:**
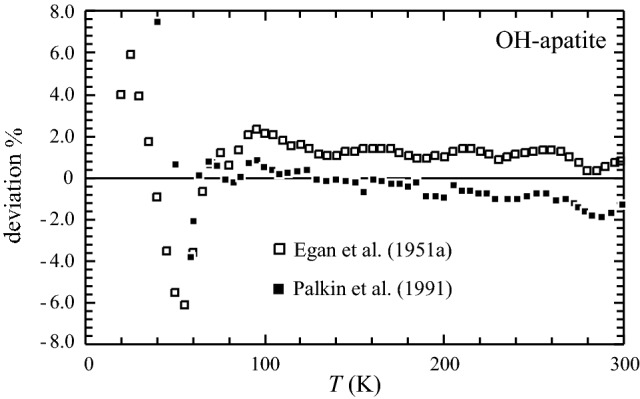
Deviation of the low-*T*AC data of Egan et al. (1951a, open squares) and Palkin et al. (1991, filled squares) from the PPMS data of OH-Ap from this study. Deviation is computed as 100 (*C*_p_^literature^—*C*_p_^PPMS^)/*C*_p_^PPMS^

**Fig. 3:**
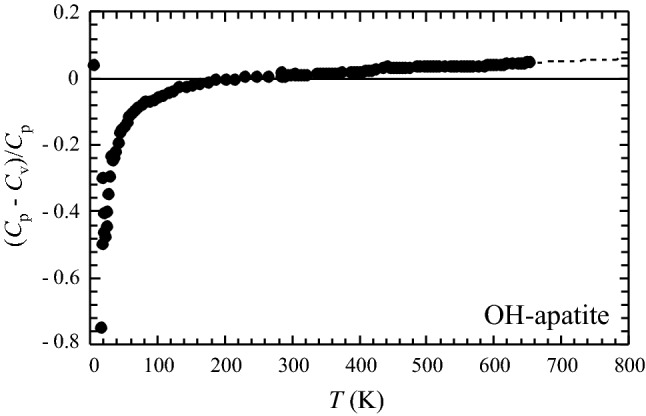
Plot of the normalised difference (*C*_p_ − *C*_v_)/*C*_p_ for OH-Ap as function of temperature. Above ca. 200 K there is a linear relationsship (Eq. 7), that was used to extrapolate heat capacities beyond 760 K (dashed line)

The PPMS- and DSC-measured heat capacities of OH-Ap are shown in Fig. 1 as a function of temperature. The PPMS data from the range 100–300 K are continuously ~ 1% smaller than the *C*_p_ data reported by Egan et al. (1951a) (Fig. 2), measured on 62 g synthetic hydroxyapatite using low-temperature adiabatic calorimetry (low-*T*AC). Below 100 K deviations are larger by up to ~ 6% in positive and negative direction, indicating some scatter in these low-temperature literature data (Fig. 2). Compared to the low-*T*AC *C*_p_ data of Palkin et al. (1991), there is good agreement to the PPMS data in the range 60 to ~ 200 K. At higher *T*’s up to room temperature, these data tend to be 1–2% smaller than PPMS-measured ones. The red dots in Fig. 1 are the heat capacity values at constant volume, *C*_v_, computed with CASTEP. The heat capacity difference *C*_v_ − *C*_p_, normalised to *C*_p_ is shown in Fig. 3. As expected, the difference *C*_p_ − *C*_v_ gets larger with rising temperature (Fig. 1), whereas (*C*_p_ − *C*_v_)/*C*_p_ approaches a linear relationship above ca. 200 K given by:

7 $$\left( {C_{{\text{p}}} {-}C_{{\text{v}}} } \right)/C_{{\text{p}}} = - 0.00{495} + {8}.0{8723}\,{1}0^{{ - {5}}} \,T\left( {\text{K}} \right)$$

**Fig. 4:**
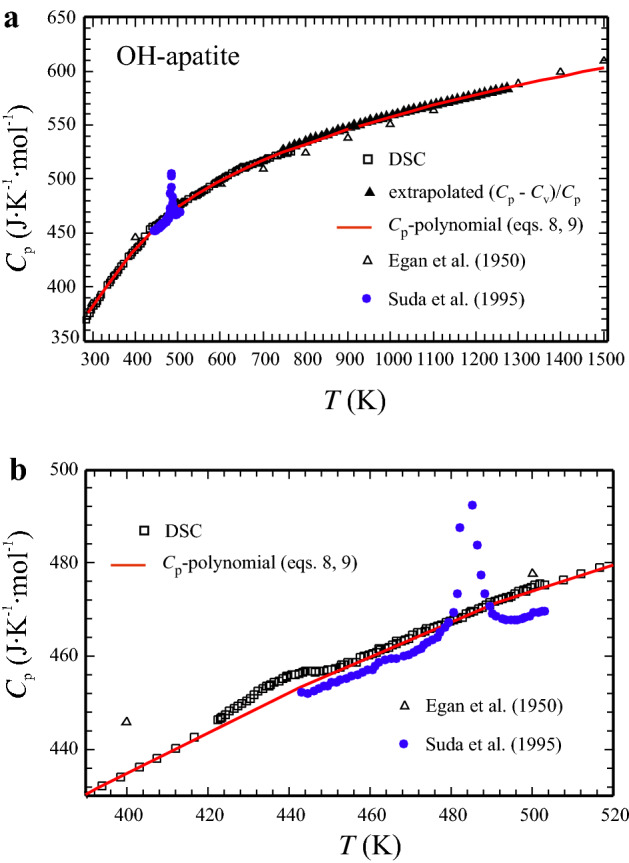
High-temperature heat capacity of OH-Ap in the temperature range **a** 300–1500 K and **b** 390–520 K. Open squares: DSC data, Filled triangles: Extrapolated *C*_p_, based on CASTEP-computed *C*_v_ and normalised (*C*_p_ − *C*_v_)/*C*_p_ as given by Eq. (7). Open triangles: Egan et al. (1951a), blue dots: Suda et al. (1995). Red curve is the *C*_p_ polynomial of Eqs. (8 and 9). Error bars are smaller than the symbol size

Following Benisek and Dachs (2018), we have used Eq. (7) to extrapolate heat capacities beyond the experimental range (760 K) based on *C*_v_ that was computed up to nearly 1300 K. This set of data, our DSC data, as well as literature *C*_p_ data are shown in Fig. 4a. The range of the structural phase transition related to OH ordering/disordering in OH-Ap is enlarged in Fig. 4b. A broad hump-shaped heat capacity anomaly in our DSC data, centred at around 442 K, is visible in this plot as a likely result of this transition. Suda et al. (1995) observed a sharper *C*_p_ peak at a higher temperature of ~ 485 K (Fig. 4b) and estimated transition enthalpy and entropy values of 630 ± 25 J mol^−1^ and 1.30 ± 0.05 J mol^−1^ K^−1^, respectively. Due to this *C*_p_ anomaly, we split our DSC data into two segments cutting out the region of the transition before fitting. The low-*T* segment (298–442 K) is given by:

8 $$\begin{aligned} C_{p} ^{{\text{OH - Ap}}} {}_{{298K - 442K}}\left( {{\text{J mol}}^{ - 1} {\text{K}}^{- 1}} \right) & = {1013.7-13735.5T^{{ - 0.5}}} \\ & \quad + 2.616718\,10^{7} T^{{ - 2}} - 3.551381\,10^{9} T^{{ - 3}} \\ \end{aligned}$$

the high-*T* segment (*T* > 442 K) by:

9 $$\begin{aligned} C_{p}^{{\text{OH - Ap}}} {}_{{ >\,442K}}\left( {{\text{J mol}}^{ - 1} {\text{K}}^{- 1}} \right) & = {877.2-11393.7\,T^{- 0.5}} \\ & \quad + {5.452030\,10^{7} \,T^{- {2}}} - {1.394125\,10^{10} \,T^{- {3}}} \\ \end{aligned}$$

Equations (8, 9) reproduce the experimental (and computed) *C*_p_ data of OH-Ap to within a mean deviation of 0.21 ± 0.16% (Fig. 4a, b, red curve). The value of *C*_p_ for OH-Ap, as derived by Egan et al. (1950) from heat-content measurements, is larger by 1–2% at room temperature up to ca. 550 K. Above that temperature, the Egan’s et al. *C*_p_ data are lower by 1.5% at maximum compared to that from this study (Fig. 4b, open triangles). Above ~ 1100 K, Eq. (9) predicts heat capacities that agree to better than 0.5% with the data of Egan et al. (1950).

Fitting the PPMS-measured *C*_p_ data on OH-Ap to Eq. (1), gives the parameters summarised in Table 1 (those for the Cl- and F-apatite endmembers are also given, as derived by Dachs et al. (2010a). Using Eq. (6), *S*^o^ of OH-Ap is then computed as *S*^o^ = 386.3 ± 2.5 J mol^−1^ K^−1^.

### Excess heat capacity and entropy of mixing along the hydroxyapatite-chlorapatite and the hydroxyapatite-fluorapatite joins

**Fig. 5:**
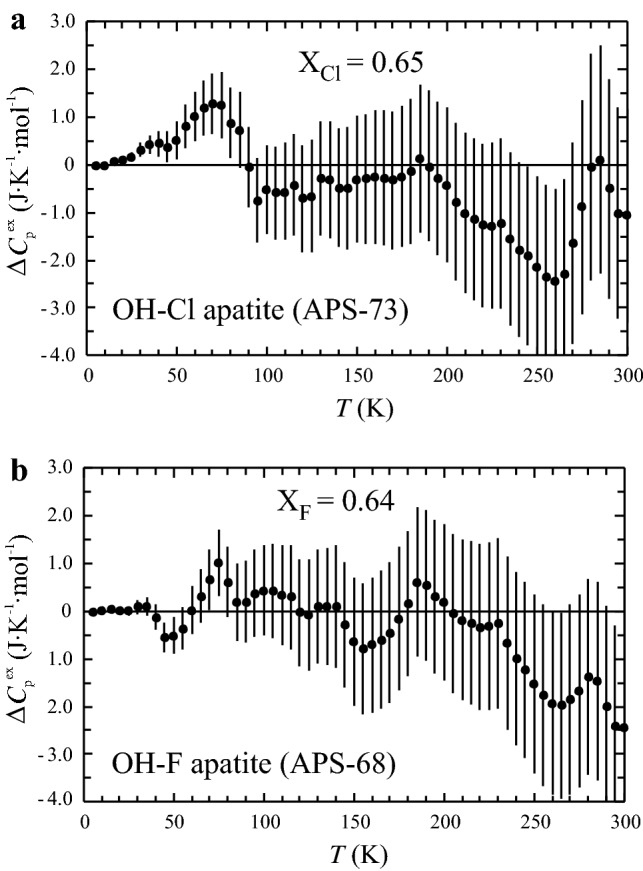
Excess heat capacity of mixing, ∆*C*_p_^ex^, as function of temperature for **a** APS-73 (X_Cl_ = 0.65) and **b** APS-68 (X_F_ = 0.67). Error bars represent ± 2σ

**Fig. 6:**
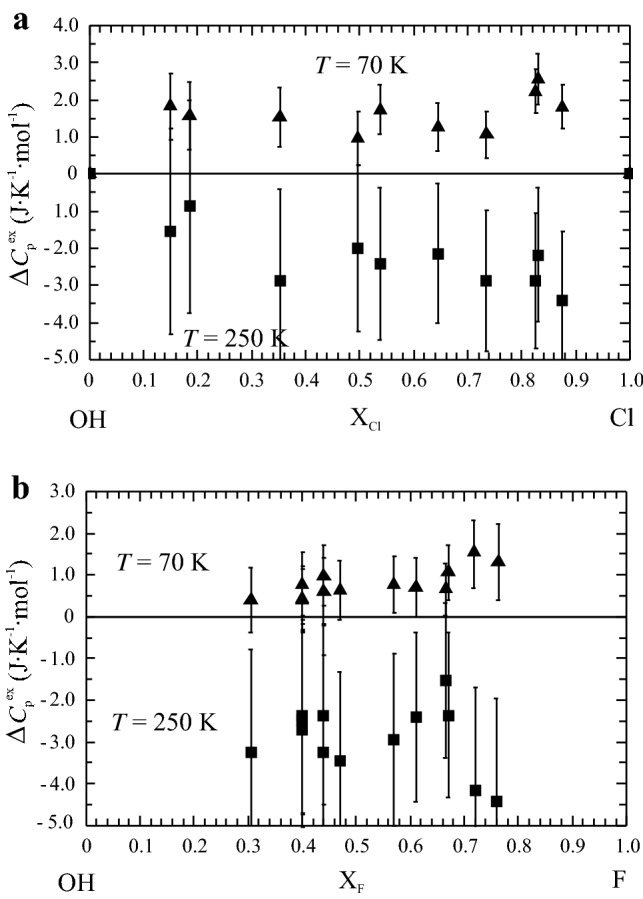
Excess heat capacity of mixing, ∆*C*_p_^ex^, for **a** OH-Cl apatites and **b** OH-F apatites at temperature sections of 70 and 250 K. Error bars represent ± 2σ

The excess heat capacity of mixing, ∆*C*_p_^ex^, was computed according to:

10 $$\Delta C_{{\text{p}}}^{{{\text{ex}}}} = C_{{\text{p}}}^{{{\text{measured}}}} {-}(C_{{\text{p}}}^{{{\text{OH}} - {\text{Ap}}}} {\text{X}}_{{{\text{OH}}}} + C_{{\text{p}}}^{{{\text{Cl}} - {\text{Ap}}}} {\text{X}}_{{{\text{Cl}}}} )$$

in the case of the OH-Ap–Cl-Ap binary join and

11 $$\Delta C_{{\text{p}}}^{{{\text{ex}}}} = C_{{\text{p}}}^{{{\text{measured}}}} {-}(C_{{\text{p}}}^{{{\text{OH}} - {\text{Ap}}}} {\text{X}}_{{{\text{OH}}}} + C_{{\text{p}}}^{{{\text{F}} - {\text{Ap}}}} {\text{X}}_{{\text{F}}} )$$

for the OH-Ap–F-Ap binary join. ∆*C*_p_^ex^, as function of temperature, is plotted in Fig. 5a, b for one representative member of each join. Similar to the Cl-Ap–F-Ap join (Dachs et al. 2010a), positive excess heat capacities related to OH-Cl and OH-F mixing in apatite occur at around 70 K in both binaries studied herein. Taking a 2σ-uncertainty into account, these are significant and larger for OH-Cl apatites amounting to 1.5–2.0 J mol^−1^ K^−1^ compared to ~ 1.0 J mol^−1^ K^−1^ for OH-F apatites. This feature of a positive ∆*C*_p_^ex^ at ~ 70 K is common in all samples and may be preceded by a small region of negative ∆*C*_p_^ex^ at 50 K in some samples (e.g., Fig. 5b). In the temperature range around 250 K, excess heat capacities tend to be generally somewhat negative (2–3 J mol^−1^ K^−1^), thus compensating for the positive ones at 70 K. At all other temperatures (including those above room temperature up to highest ones), ∆*C*_p_^ex^ is zero within a 2σ-uncertainty. Figure 6 shows ∆*C*_p_^ex^ as function of composition at temperature sections of 70 and 250 K, illustrating the ∆*C*_p_^ex^ behaviour as discussed above.

**Fig. 7:**
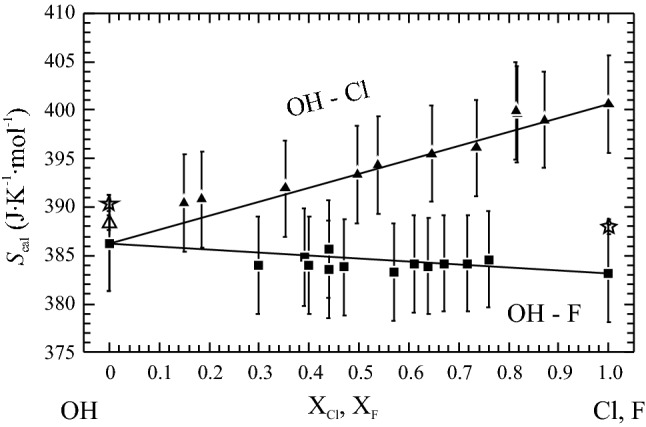
Calorimetric entropies, *S*_cal_, at *T* = 298.15 K as function of composition for the OH-Ap–Cl-Ap binary (filled triangles) and the OH-Ap–F-Ap binary (filled squares). Data are from Table 2. Straight lines indicate ideal mixing behaviour. Open stars are *S*^o^ of the OH- and F-Ap endmembers according to Egan et al. (1951a; b), open triangle is *S*^o^ of OH-Ap reported in Palkin et al. (1991). Error bars represent ± 2σ

A plot of *S*_cal_ at 298.15 K for OH-Cl and OH-F apatites (Table 2), as a function of composition, indicates linear behaviour within error (Fig. 7). There are thus no excess entropies of mixing in these solid solution apatites.

**Table 2:** Calorimetric entropy, *S*_cal_, at 298.15 K of members of the OH-F and OH-Cl apatite binaries

OH-F apatite	X_F_	*S*_cal_ J mol^−1^ K^−1^	OH-Cl apatite	X_Cl_	*S*_cal_ J mol^−1^ K^−1^
OH–Ap	0.000	386.3	OH-Ap	0.000	386.3
APS92	0.298	384.0	APS94	0.150	390.4
APS91	0.390	384.8	APS93	0.185	390.8
APS77	0.400	384.0	APS80	0.352	391.9
APS70	0.440	383.6	APS71	0.497	393.4
APS85	0.440	385.7	APS74	0.538	394.3
APS69	0.470	383.8	APS73	0.645	395.5
APS84	0.570	383.3	APS72	0.735	396.1
APS86	0.610	384.1	APS78	0.817	399.6
APS68	0.639	383.9	APS82	0.814	399.9
APS87	0.670	384.2	APS83	0.871	399.0
APS95	0.718	384.2			
APS96	0.759	384.6			
F-Ap	0.999	383.2	Cl-Ap	0.998	400.6

X_F_ and X_Cl_ are calculated as F/(F + OH) and Cl/(Cl + OH), respectively. Data are plotted in Fig. 7. The 1σ-uncertainty of the *S*_cal_ data is ± 2.5 J mol^−1^ K^−1^

## Discussion

Surprisingly, even though OH-Ap is probably the most important apatite end member (at least in the biosciences), there are only the two studies from Egan et al. (1951a) and Palkin et al. (1991) that provide low-*T* heat capacity data on OH-Ap. From our PPMS data we derived *S*^o^ = 386.3 ± 2.5 J mol^−1^ K^−1^ for this phase, whereas Egan et al. (1951a) computed *S*^o^ = 390.4 ± 0.4 J mol^−1^ K^−1^ from their low-*T*AC data. The PPMS-derived *S*^o^ for OH-Ap in this study, in combination with *S*_cal_ along the OH-Cl and OH-F apatite binaries (Table 2) and *S*^o^ for Cl- and F apatite from Dachs et al. (2010a), gives a consistent picture of an ideal OH-Cl and an ideal OH-F mixing behaviour for these binaries as was the case for the Cl-F binary. This may be taken as an indication that the value *S*^o^_OH-Ap_ = 386.3 ± 2.5 J mol^−1^ K^−1^ from this study should be reliable. Egan et al. (1951a) used 62 g from two samples in their low-*T*AC measurements that were synthesized using an acidimetric precipitation method. In one sample (Hy-43) two weak lines corresponding to the two strongest lines of Cl-Ap were found in the XRD pattern of this OH-Ap. In the synthesis routine of the other sample (XP-12), a Cl-bearing substance, namely ammonium chloride was definitely added to promote crystal growth. The ~ 1% larger entropy value of OH-Ap reported by Egan et al. (1951a) could thus be explained by supposing that some Cl might have replaced OH in their ‘OH-apatite’. Based on Fig. 7, ~ 20% Cl substituting for OH would suffice to increase the entropy of such an OH-Cl apatite to the value measured by Egan et al. (1951a). Palkin et al. (1991), on the other hand, report a *S*^o^ of 388.4 ± 1.6 J mol^−1^ K^−1^ for OH-Ap which agrees within error with that from this study (Fig. 7).

We interpret the hump-shaped heat capacity anomaly that we observe in our DSC data of OH-Ap (APS-88) around 442 K to be caused by the monoclinic to hexagonal phase transition related to OH ordering/disordering (van Rees et al. 1973; Suda et al. 1995). Suda et al. reported a sharper *C*_p_ anomaly at a higher temperature of ~ 485 K. The reason for this discrepancy is not clear but may have to do with the concentration of vacancies on hydroxyl sites in the OH-Ap. Kijima and Tsutsumi (1979) sintered solution grown crystals of hydroxyapatite at temperatures between 1050 °C and 1450 °C to dense polycrystalline bodies. They used a Laser flash method to measure heat capacity, thermal diffusivity and conductivity of these aggregates, but did not observe any *C*_p_ anomalies. As they note, the sintering process is accompanied by the formation of vacancies due to thermal dehydration so that their samples were oxyhydroxyapatites lacking OH-groups in their structure. Because the OH-Ap studied herein (APS-88) was crystallised in excess of H_2_O at 400 MPa/1000 °C (72 h) in a sealed Pt capsule, hydroxyl-site vacancies should play an only minor role if any in the OH-Ap from this study. We have, however, no reasonable explanation why the *C*_p_ anomaly measured in our hydroxapatite occurs at a lower temperature and is not as sharp as observed by Suda et al. (1995) in their synthetic OH-Ap sample.

**Fig. 8:**
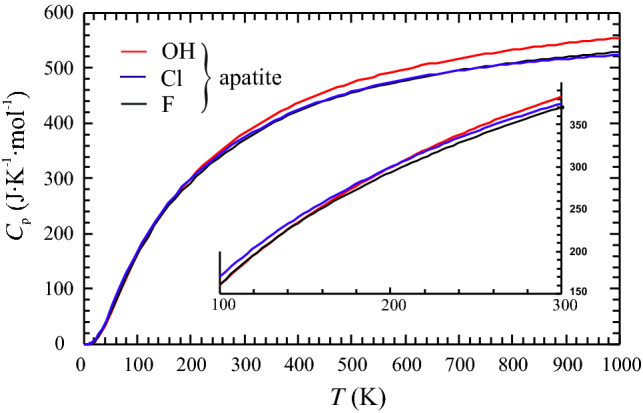
Heat capacity of the OH-, Cl- and F-endmembers as function of temperature between 0 and 1000 K. *C*_p_ of OH-Ap (red) was computed according to this study (Table 1, Eqs. 8, 9), *C*_p_ of Cl- and F-Ap (blue and black curves) following Dachs et al. (2010a). See text for further discussion

The heat capacity behaviour of the three apatite endmembers, OH-Ap, Cl-Ap and F-Ap is shown from 0 to 1000 K in Fig. 8. As a function of temperature, the *C*_p_ curves of Cl- and F-Ap run continuously subparallel. The lattice vibrational behaviour of these two endmembers is thus quite similar and the 5–10 J mol^−1^ K^−1^ larger heat capacity of Cl-Ap is simply a mass effect, with the atomic mass of Cl being roughly double that of F. In contrast, the *C*_p_ curve of OH-Ap, is the lowest at *T* < 100 K, intersects both other curves between ca. 100 and 200 K, and then remains the highest curve with increasing temperature. At *T* = 1000 K, the difference to the *C*_p_ curves of the two other endmembers is 25–30 J mol^−1^ K^−1^. As discussed in Dachs et al. (2010b), similar heat capacity behaviours, i.e., *C*_p_^OH−endmember^ < *C*_p_^F−endmember^ at low *T* and vice versa at high *T*, with a crossover between 50 and 180 K, were observed for OH/F-pargasite, OH/F-phlogopite, and Mg(OH)_2_/MgF_2_. These authors gave the following lattice dynamic interpretation for that. Namely, since the bonds of the octahedral cations with oxygen are stronger than the corresponding ones with F, the frequencies of the cation-O vibrations in the OH-endmember will be higher than in the F or Cl counterpart, causing the observed heat capacity behaviour at low temperatures (lower *C*_p_ of the OH-endmember compared to the F- or Cl-endmember). The OH group, however, has additional vibrational degrees of freedom (e.g., OH-liberation modes in the range 600–800 cm^−1^, Freund and Knobel 1977; Rintoul et al. 2007), that become increasingly excited with rising *T*, providing a contribution to *C*_p_ of the OH-endmember that is missing in the F- or Cl-endmember. As a result, *C*_p_^OH−endmember^ becomes larger at some intermediate temperature than *C*_p_^F(or Cl−)endmember^.

The extrapolation of the heat capacity from OH-Ap, beyond the experimental range (760 K) based on the CASTEP-computed *C*_v_ and linear extrapolation of the normalised difference (*C*_p_ − *C*_v_)/*C*_p_, as given in Eq. (7), yields values that are in good agreement with the *C*_p_ derived by Egan et al. (1950) from heat-content measurements above 1000 K (Fig. 4). This confirms that the *C*_p_ polynomial for OH-Ap in this study (Eq. 9) can be used reliably up to 1500 K to compute the heat capacity of OH-Ap.

In both apatite joins studied herein, there are small but significant positive excess heat capacities of mixing at low *T* around 70 K. These are compensated by slightly negative ones at around 250 K, so that, similar to the situation found for the Cl-F apatites (Dachs et al. 2010a), there is ideal entropic OH-Cl and OH-F mixing behaviour in apatite at 298.15 K.

## Supplementary Information

Below is the link to the electronic supplementary material.

Supplementary file1 (XLS 224 KB)

## Data Availability

All calorimetric data are given in Supplementary Table 1, other data in Tables 1 and 2.
